# Insights into the Antennal Characteristics and Olfactory Strategy of the Endangered Rhino Stomach Bot Fly *Gyrostigma rhinocerontis* (Diptera: Oestridae)

**DOI:** 10.3390/insects13100889

**Published:** 2022-09-29

**Authors:** Wentian Xu, Xinyu Li, Qike Wang, Chenglin Zhang, Minghai Yang, Tongshan Zhou, Kai Li, Dong Zhang

**Affiliations:** 1 School of Ecology and Nature Conservation, Beijing Forestry University, Qinghua East Road 35, Beijing 100083, China; 2 School of BioSciences, University of Melbourne, Melbourne, VIC 3010, Australia; 3 Beijing Zoo, Beijing 100044, China; 4 Beijing Key Laboratory of Captive Wildlife Technologies, Beijing Zoo, Beijing 100044, China; 5 Yantai City Garden Construction and Maintenance Center, Yantai 264000, China

**Keywords:** *Gyrostigma rhinocerontis*, antenna, morphology, myiasis, parasite, pedicel, sensilla, sensory pit, ultrastructure

## Abstract

**Simple Summary:**

*Gyrostigma rhinocerontis* (Hope), the rhinoceros bot fly, is a rare obligate intestinal parasite of white and black rhinoceroses that can cause severe myiasis and secondary infection, leading to enormous economic and scientific loss. As the main sensory organs of flies, the antennae provide insects with critical information about the environment, playing significant roles in their key activities. The antennal characteristics of *G. rhinocerontis* remain largely unexplored, probably due to the extreme rarity of adult specimens in collections. In this study, the antennae of *G. rhinocerontis* were thoroughly examined using light and scanning electron microscopy. The morphology, including detailed ultrastructure, of antennal sensilla are presented. As the largest species of Oestridae Leach, not surprisingly, *G. rhinocerontis* has significantly larger antennae with more sensilla and sensory pits than any other Oestridae species, which could be an adaptation to locate their rare and endangered hosts.

**Abstract:**

*Gyrostigma rhinocerontis* (Diptera: Oestridae) is a rare obligate intestinal parasite of both white and black rhinoceroses, which can induce severe myiasis, cause secondary infection, and lead to enormous economic and scientific loss. Antennae are the main sensory organs of *G. rhinocerontis*, which may have evolved a series of specialized adaptive structures to facilitate the exploitation of their hosts. Here, we thoroughly examine the antennae of *G. rhinocerontis* via light and scanning electron microscopy. Only microtrichia and chaetic sensilla were observed on the scape and pedicel, and the latter is enlarged, half-enveloping the postpedicel. Four types of sensilla (trichoid sensilla, basiconic sensilla, coeloconic sensilla, and clavate sensilla) and sensory pits are detected on the postpedicel. A set of coeloconic sensilla and a chaetic sensillum are located on the arista. Distribution, type, size, and ultrastructure of antennal sensilla are presented. The antennae of *G. rhinocerontis* are the largest among Oestridae species, with the most sensilla and the most sensory pits. These antennal characteristics could be correlated to their adaptation for more sensitive and accurate olfactory organs, used to locate their rare and endangered hosts. Accordingly, this morphological evidence supports that the host is an important driving factor in the diversity of antennal morphology in the bot flies.

## 1. Introduction

Parasitic flies (e.g., bot fly, flesh fly, blow fly, latrine fly, etc.) are of great medical and veterinary importance, as they are predominantly responsible for severe myiasis and secondary bacterial infection [1,2]. These infections often cause mortality in domestic and wild animals (many of them endangered), and even in humans [3,4,5,6,7,8,9]. An astonishing feature of these parasitic flies is their ability to detect host-derived chemical cues [10,11,12], which allows the obligate parasitic bot flies (i.e., Oestridae Leach), to locate their specific and sometimes rare hosts at great distances within their short adult life span (around 3–10 days) [4,9].

*Gyrostigma rhinocerontis* (Hope) (Oestridae: Gasterophilinae) is a rare obligate parasite adapted to larval life in the alimentary canal of both white (*Ceratotherium simum* (Burchell)) and black rhinoceroses (*Diceros bicornis* Linnaeus) [4,7,9,13]. These flies can cause severe gastrointestinal myiasis [14,15,16] and may be detrimental to the health of an individual rhinoceros and to their population’s viability. As a high proportion of both the white and black rhinoceros populations are bred from small populations, high too are their chances of inbreeding and genetic defects [17]. For example, the entire population of the Southern white rhinoceros was brought from fewer than 50 to about 17,000–18,000 [18], making them potentially vulnerable to parasites and diseases. This resembles the similar case of Przewalski’s horse (*Equus przewalskii* Poliakov), which is currently threatened by the parasitic horse bot flies that have killed up to 75% of first-year foals [19,20]. On the other hand, despite the conservation success, the population of the black rhinoceros in Africa is still critically small (around 5500, as estimated by World Wild Life in 2020; www.worldwildlife.org, 5 July 2022), and the population of the Southern white rhinoceros has declined over the past years because of poaching [17], suggesting that their obligate parasite *G. rhinocerontis* is also rare and potentially endangered [13,21]. Nevertheless, the biology of *G. rhinocerontis* is poorly understood despite their potential impact on the conservation management strategies of rhinoceroses in Africa.

The ultrastructure of genus *Gyrostigma* Brauer antennae has rarely been documented. As the most important olfactory sensory organs where the majority of chemical receptors are located (e.g., [10,11,12]), the antennae are shaped by the host type, behaviour, biology, and the exploitation of the flies [11,22,23]. Bot flies were suggested to possess a broad range of antennal diversities and adaptations [24,25,26,27,28,29]. In the subfamily Gasterophilinae, the antennal structure of six out of eight species of *Gasterophilus* Leach have been thoroughly studied so far [25,26]. However, there is a paucity of reports on the remaining two genera (i.e., *Gyrostigma* and *Cobboldia* Brauer), mostly due to the extreme scarcity of specimens across the globe, as they are the obligate parasites in the alimentary tract of rhinoceroses and elephants, respectively [4,7,8,9]. These data could provide valuable information for understanding the structural antenna adaptation in parasitic flies and their host–parasite co-evolutionary relationships.

This study focuses on the antennae of *G. rhinocerontis*, illustrates all antennal segments in detail using light and scanning electron microscopy, and describes and summarises the type, size, and morphological characteristics of the antennae and the sensilla. Finally, we compare the antennal morphology among bot flies and explore their adaptive characteristics and olfactory strategies for parasitism.

## 2. Materials and Methods

### 2.1. Acquisition of Samples

Among different families in calyptrate species, males and females usually have morphologically similar antennae and the same (sub) types of sensilla; the females possess more numerous and denser sensilla on the postpedicel [25,26,27,28,29,30,31,32,33,34,35,36,37]. The adult *G. rhinocerontis* specimens used in this study were reared from third instar larvae naturally located in the feces of a white rhino (*Ceratotherium simum*), which was legally imported from South Africa in 2015 and housed in the Beijing Zoo, Beijing, China. The mature third instars were immediately transferred to an artificial climate box for 5 weeks of incubation under 50% humidity, at 25 °C in the day and 14 °C at night. The specimens were identified, pinned as museum samples, and deposited in the Beijing Key Laboratory of Captive Wildlife Technologies, Beijing Zoo. All the mounted samples were saved as voucher specimens numbered as GyroI–V. Due to the extreme rarity of *G. rhinocerontis* specimens in collections [16,38], a total of five specimens were examined in the present study (2 females, 3 males). Among them, antennae of a female and a male were imaged in detail.

### 2.2. Morphological Analyses under Light and Scanning Electron Microscopy

The external morphology of antennae was observed using an Olympus SZX16 stereoscopic microscope (Olympus Corp., Tokyo, Japan). Series photographs were taken with a Canon 600D digital camera (Canon, Inc., Tokyo, Japan), mounted on the stereoscopic microscope, and superimposed by Helicon Focus. Then, all micrographs were processed on Windows 10 platform using Adobe Photoshop CS6 (Adobe Systems, Inc., San Jose, CA, USA).

For scanning electron microscopy (SEM) observation, the antennae of a female were excised under the stereoscopic microscope, immersed in phosphate-buffered saline (PBS) buffer (pH 7.4), and cleaned with detergent solution via ultrasonic cleaner (3 times, 5 min each). Subsequently, samples were rinsed twice in normal saline solution and dehydrated in an ascending ethanol series (15 min each with 60, 70, 80, 90, 95, and twice in 100% ethanol) and soaked twice (10 min each) in hexamethyldisilazane (HMDS) followed by vacuum-drying. The female antennae were mounted on aluminum stubs with conductive adhesives and left in a desiccator overnight to dry thoroughly, then coated with gold and examined using a HITACHI SU8010 SEM (Hitachi Corp., Tokyo, Japan). To keep the male specimen intact, the entire body of the male was mounted on an aluminum stub without dissecting and coating. The body and the antennal postpedicel length of two females and three males were measured. The length, basal diameter, and distal dilation diameter of five types of female antennal sensilla were measured (*n* = 15 for each type of sensilla).

The sensilla were described and classified according to Zhang et al. [25,26]. The morphometric data of the antennal postpedicel and sensilla were modified from Cumming and Wood [39].

## 3. Results

### 3.1. General Description of the Antenna in G. rhinocerontis

**Figure 1:**
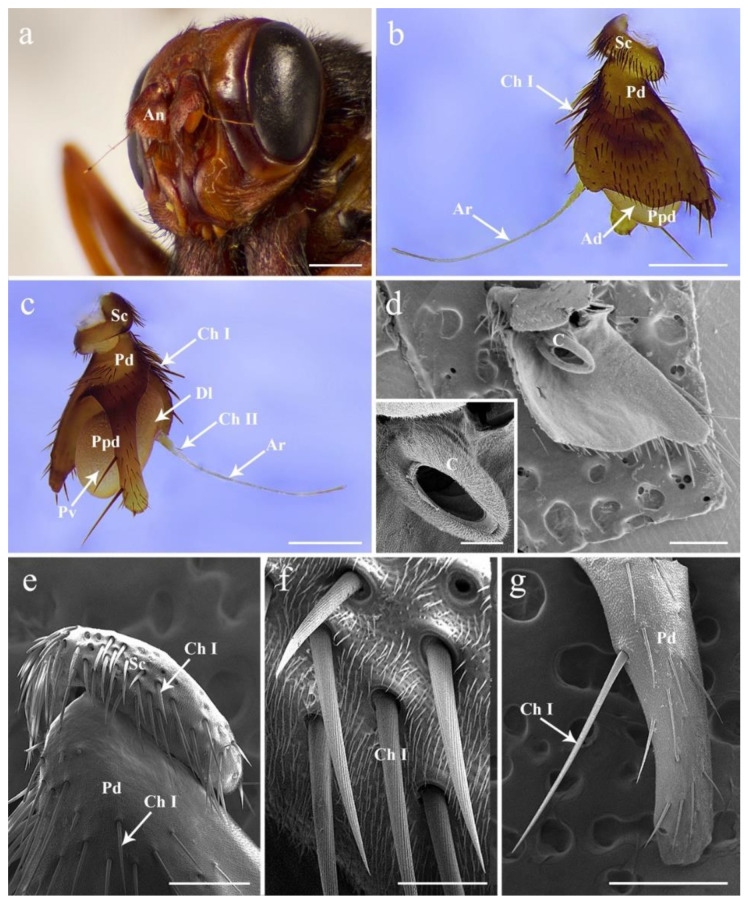
General morphology of antenna in female *Gyrostigma rhinocerontis*. (**a**) Antennae located centrally between compound eyes in resting position. (**b**) Anterodorsal surface of antenna. (**c**) Dorsolateral and posteroventral surface of antenna. (**d**) Details of antennal pedicel after removal of the postpedicel. (**e**) Anterodorsal surface of antennal scape showing the distribution of chaetic sensilla I. (**f**) Chaetic sensilla I on antennal scape showing the longitudinal grooves. (**g**) Ventral surface of the pedicel. Scale bars: (**a**) = 2 mm, (**b**,**c**) = 1 mm, (**d**) = 500 μm, 15 μm in inset, (**e**) = 250 μm, (**f**) = 50 μm, (**g**) = 500 μm. Abbreviations: Ad, anterodorsal surface; An, antenna; Ar, arista; C, conus; Dl, dorsolateral surface; Ch I, chaetic sensillum I; Ch I, chaetic sensillum II; Pd, pedicel; Ppd, postpedicel; Pv, posteroventral surface; Sc, scape.

Both males and females of *G. rhinocerontis* show a similar arrangement of the antennal pattern, with a pair of segmented sensory appendages located on the frontal region of the head between the compound eyes and below the lunule (Figure 1a). The antennae are composed of three segments: proximal scape (Sc), pedicel (Pd), and distal flagellum consisting of a pyriform postpedicel (Ppd) and a long, tapered, and slender arista (Ar) extending laterally (Figure 1b,c). No sexual dimorphism has been observed in the antennal structures.

### 3.2. Antennal Scape and Pedicel

The scape is the shortest and most proximal segment (Figure 1a–c,e). The pedicel is enlarged, half envelopes the postpedicel, and deeply splits into two parts: the dorsal part is approximately triangular and hides the postpedicel, while the ventral part is elongated, projecting below the ventral surface of the antennal postpedicel (Figure 1a–d,g). Both scape and pedicel are ornamented with dense, hair-like microtrichia, and sparse chaetic sensilla I (Ch I) (Figure 1). The Ch I are acuminate, strong, and relatively straight, with twisted longitudinal grooves on the cuticular surface, inserted into indistinct sockets and varying in length (Figure 1e–g). The distal part of the pedicel is composed of the conus and an annular ridge that can be examined after removing the postpedicel (Figure 1d). No pedicellar button was observed on the articular surfaces between pedicel and postpedicel.

### 3.3. Antennal Postpedicel

**Figure 2:**
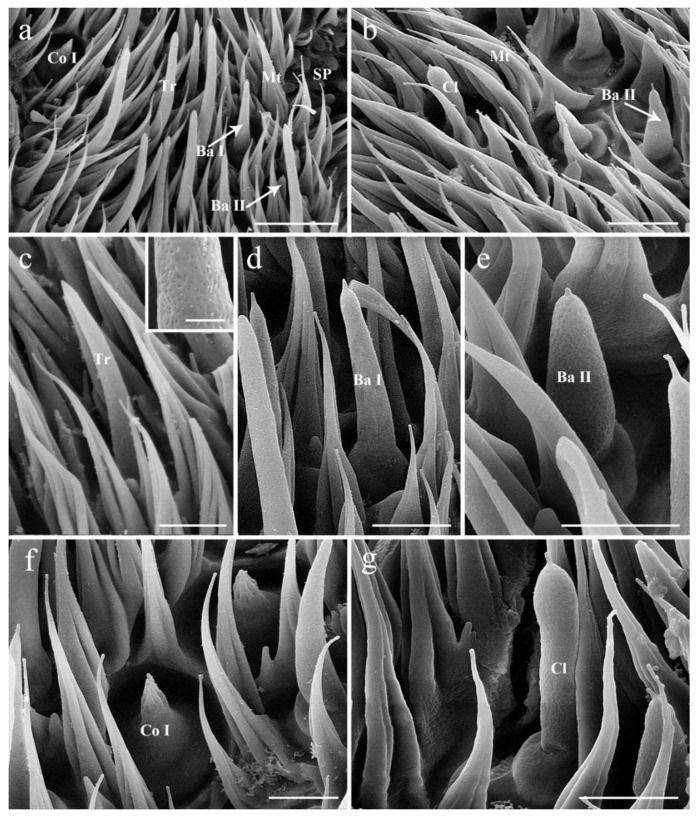
Scanning electron micrographs of the sensilla on postpedicel in female *Gyrostigma rhinocerontis*. (**a**,**b**) Distribution of sensilla on the postpedicel surface. (**c**) Trichoid sensillum, showing numerous pores on surface in box. (**d**) Basiconic sensilla I. (**e**) Basiconic sensilla II with cuticular wall pierced by obvious pores. (**f**) Coeloconic sensillum I, set in shallow depression on postpedicel surface. (**g**) Clavate sensillum penetrated by numerous pores on cuticular wall. Scale bars: (**a**) = 15 μm, (**b**) = 10 μm, (**c**–**g**) = 5 μm. Abbreviations: Ba I, basiconic sensillum I; Ba II, basiconic sensillum II; Co I, coeloconic sensillum I; Cl, clavate sensillum; Mt, microtrichia; SP, sensory pit; Tr, trichoid sensillum.

**Figure 3:**
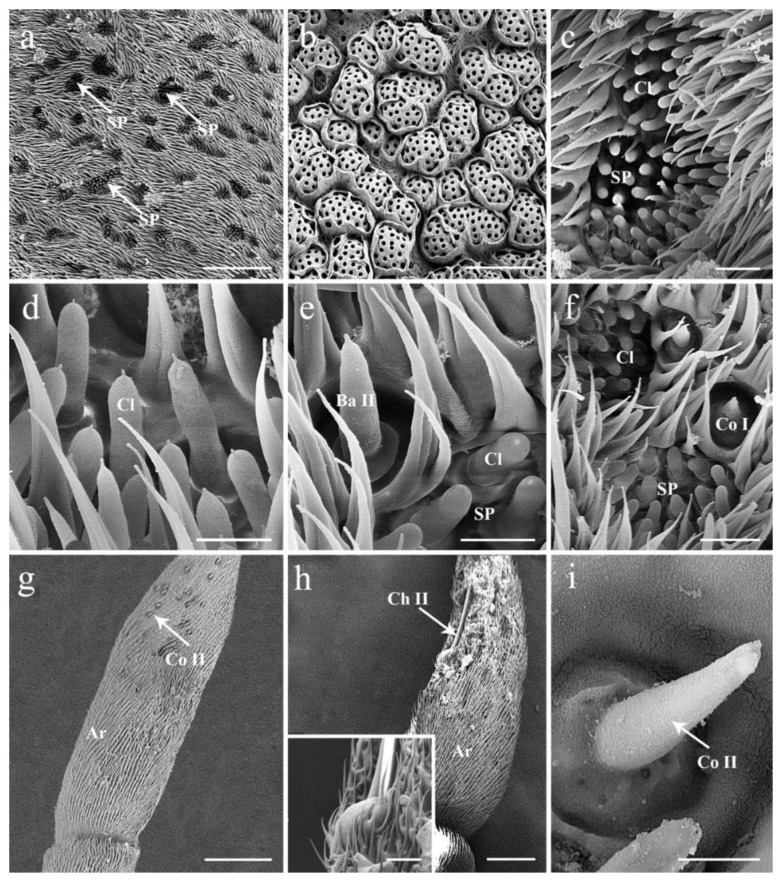
Scanning electron micrographs of sensory pits and the arista in female *Gyrostigma rhinocerontis*. (**a**) A large amount of sensory pits on postpedicel surface. (**b**) Interior surface of sensory pits, showing fusion between adjacent pits. (**c**) Dorsal view of ‘super sensory pit’. (**d**) Clavate sensilla clustered in a sensory pit. (**e**) Sensory pit accompanied by single basiconic sensillum. (**f**) Sensory pit accompanied by single coeloconic sensillum. (**g**) Coeloconic sensilla II on basal part of second segment of arista. (**h**) Chaetic sensillum II on basal part of arista, with swelling at base (insert). (**i**) Magnification of coeloconic sensilla II in *G. rhinocerontis* Scale bars: (**a**,**g**) = 100 μm, (**b**) = 50 μm, (**c**,**f**) = 10 μm, (**d**,**e**) = 5 μm, (**h**) = 50 μm, 10 μm in inset, (**i**) = 3μm. Abbreviations: Ar, arista; Ba II, basiconic sensillum II; Co I, coeloconic sensillum I; Co II, coeloconic sensillum II; Cl, clavate sensillum; Ch II, Chaetic sensillum II; SP, sensory pit.

**Figure 4:**
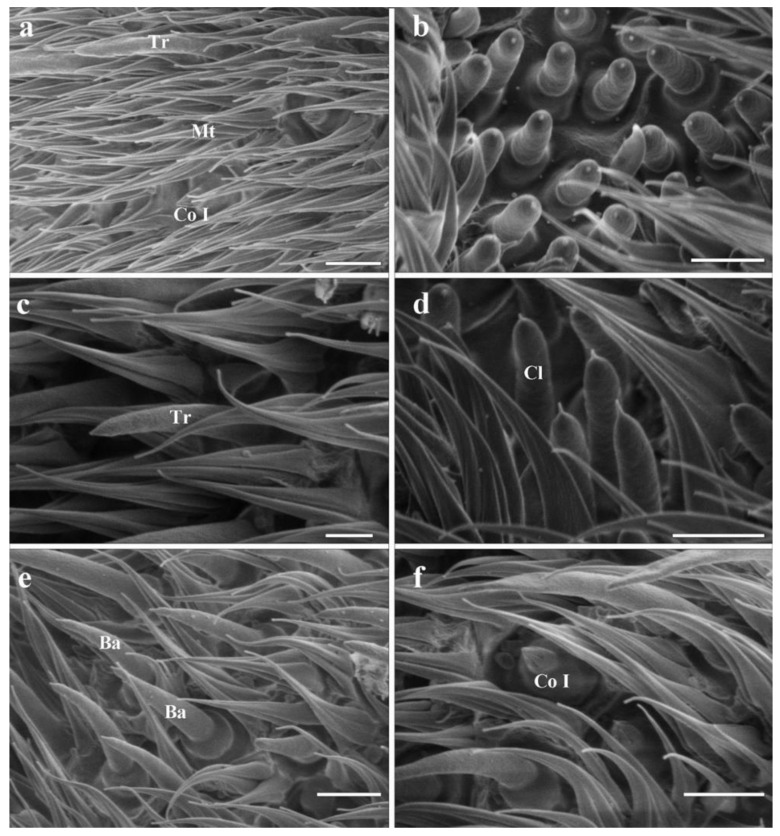
Scanning electron micrographs of sensilla on the postpedicel in male *Gyrostigma rhinocerontis*. (**a**) Distribution of sensilla on postpedicel surface. (**b**) Dorsal view of ‘super sensory pit’. (**c**) Trichoid sensillum. (**d**) Clavate sensilla clustered in sensory pit. (**e**) Basiconic sensilla. (**f**) Coeloconic sensillum I, set in shallow depression on postpedicel surface. Scale bars: (**a**,**b**,**d**–**f**) = 5 μm, (**c**) = 2.5 μm. Abbreviations: Ba, basiconic sensillum; Co I, coeloconic sensillum I; Cl, clavate sensillum; SP, sensory pit; Tr, trichoid sensillum.

The postpedicel (1.56–1.91 mm in female, 1.64–1.86 mm in male) is the most prominent part possessing numerous and diverse sensilla. It can be divided into three aspects: the anterodorsal surface (Ad), the dorsolateral surface (with arista) (Dl), and the posteroventral surface (Pv) (Figure 1b,c). In total, four types of sensilla are detected in both sexes (Figure 2, Figure 3 and Figure 4), including trichoid sensilla (Tr) (Figure 2a,c), basiconic sensilla (Ba) (Figure 2a,b,d,e), coeloconic sensilla (Co) (Figure 2a,f), and clavate sensilla (Cl) (Figure 2b,g and Figure 3c–f). A large number of sensory pits (SP) are observed on the surface (Figure 3a,c,d). The microtrichia on the postpedicel are flat and grooved (Figure 2 and Figure 3e), with multiple micropores basally (Figure 2e,g and Figure 3e). The arista (Ar) is attached to the dorsolateral surface of the postpedicel (Figure 1a–c). It consists of a short basal and a long distal segment that are ornamented with short hair-like microtrichia. A set of coeloconic sensilla II (Co II) (Figure 3g,i) and a chaetic sensillum II (Ch II) (Figure 1c and Figure 3h) are located on the basal part of the distal segment. Ch II possesses an obvious basal bulb, without distinct cuticular grooves.

### 3.4. General Description of the Sensilla on Postpedicel

#### 3.4.1. Trichoid Sensilla

Trichoid sensilla (Tr) are the longest among the four types (Table 1 and Figure 4). Each sensilla is slender, elongated, and multiporous, extending above the microtrichia, and gradually tapered to a blunt tip (Figure 2a,c). Trichoid sensilla are concentrated spread on the anterodorsal surface and show a conspicuous density gradient, with the number increasing from the basal to the apical part of the postpedicel.

#### 3.4.2. Basiconic Sensilla

Basiconic sensilla (Ba) are digitiform, arise from conspicuously swollen bases, and abruptly taper into blunt tips, with cuticular walls pierced by distinct pores from base to top (Figure 2a,b,d,e). Two subtypes (Ba I, II) are summarized based on their shape and size (Table 1) in females. The basiconic sensilla II are shorter and broader than basiconic sensilla I (Figure 2d,e and Table 1). The absence of basiconic sensilla II in males may be due to the inability to dissect the specimen, and thus to capture more area, during SEM work.

#### 3.4.3. Coeloconic Sensilla

Coeloconic sensilla (Co) are the shortest apparatus of all the sensilla (Table 1 and Figure 4), and can be divided into two subtypes (Co I, II) according to their morphology and distribution (Figure 2a,f). Coeloconic sensilla I are seated in sunken cavities on the surface of the postpedicel, characterized by distinct longitudinal ridges on their cuticular walls (Figure 2f). They are randomly scattered and often hidden by surrounding microtrichia. Coeloconic sensilla II are only distributed on the arista, in shallow depressions at the base of the distal aristal segment (Figure 3g,i). They are longer than coeloconic sensilla I, with smooth cuticle.

#### 3.4.4. Clavate Sensilla

Clavate sensilla (Cl) are the most numerous sensilla in *G. rhinocerontis*. They are featured by subapical dilatation or swelling, giving them a club-like or spatulate appearance (Figure 2b–g, Figure 3c–f and Figure 4, Table 1). Every clavate sensillum arises from a swollen bulb, decorated with a rather abrupt tip on the top, and penetrated by a large number of pores on the cuticle. Clavate sensilla are detected mainly in sensory pits, while only a few of them are singly distributed in a superficial cavity on the surface of the postpedicel (Figure 2g and Figure 3c,d).

#### 3.4.5. Sensory Pits

The sensory pit (SP) is characterized by a cave-like depression clustered with several sensilla of the same type on the surface of the postpedicel. Numerous sensory pits (about 300) are widely spread on the postpedicel surface, especially on the proximal part (Figure 3a and Figure 4); and only clavate sensilla are detected in them (Figure 3c–f). Adjacent sensory pits were fused in many cases and can be recognized from both external and internal surfaces (Figure 3b,f). These pit fusions or combination makes them into a ‘super sensory pit’ that contains double or triple or more clustered sensilla (Figure 3a–c). A single basiconic sensillum or coeloconic sensillum is often found accompanying the ‘super sensory pit’ closely on the side (Figure 3e,f).

## 4. Discussion

**Figure 5:**
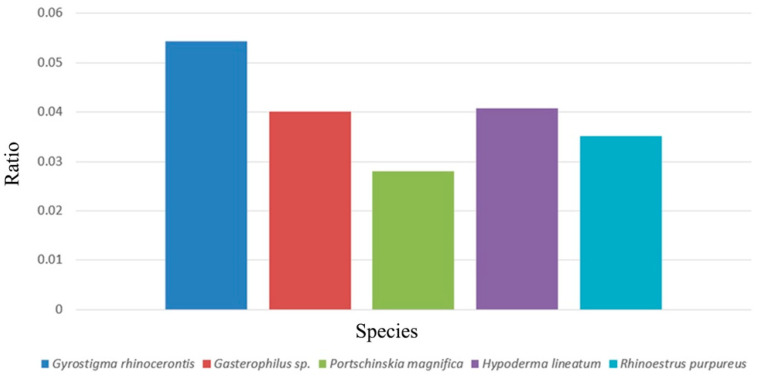
Length ratio of postpedicel and body (ratio = length of postpedicel/length of body) in *G. rhinocerontis*, *Gasterophilus* species, *Portschinskia magnifica*, *Hypoderma lineatum,* and *Rhinoestrus purpureus* (using modified data from [25,26,27,28,34]).

This study demonstrates the unique antennal characters of the rhinoceros bot fly *G. rhinocerontis*, a rare obligate intestinal parasite of the endangered white and black rhinoceros. As the largest fly species in Africa, with a body length reaching 24–41 mm [4,7,16], *G. rhinocerontis* is an exceptionally large species with exceptionally large antennae (Supplementary File S1). Compared with other Oestridae species (e.g., *P*. *magnifica*, *Gasterophilus* spp., *H*. *lineatum* and *R*. *purpureus*), whose body lengths are only 1/4–2/3 of *G. rhinocerontis* [4,7,9], the antennal postpedicel of *G. rhinocerontis* is the largest both in absolute and relative length (Figure 5). The ratio of postpedicel and body length of *G. rhinocerontis* is expectedly the highest (around 0.054), whereas others are around 0.028–0.038 (Figure 5). The length of its postpedicel is around 1.8 mm, which is significantly longer than any other Oestridae species, and is one of the longest in comparison to most other phytophagous (~0.27 mm) [33], predatory (~0.35 mm) [40], saprophagous (~0.38 mm) [37], and parasitic (~1.2 mm) [41,42] Calyptrate species. The large antennal size may reflect the high demand for the acute olfactory senses to locate suitable mates and their sparsely distributed hosts in their exceptionally short lifespan (merely 3–5 days) [4,7,9]. The exact olfactory cues for *G. rhinocerontis* to locate their mates and their specific hosts remain unknown, but the sheer antennal size of this species may reflect the selection pressure for acquiring these odours. Anecdotal records showed that white and black rhinos are usually solitary or gregarious in small herds amongst large numbers of other herbivores and can cover vast areas in a day [43], making tacking them extremely difficult. Olfactory organs are one of the most demanding animal tissues, in terms of the development and energy consumption required for maintaining their function [44]. *Gyrostigma rhinocerontis* might have evolved the larger postpedicel at high costs to increase their chances to locate their hosts.

The shape of pedicel in *G. rhinocerontis* is unique compared to the vast majority of other calyptrate species [20,24,29,30,31,32,33,34,35,36]. The pedicel is the second segment of the antenna, which is usually distinctly shorter than the postpedicel. As a potential diagnostic character for species identification, the pedicel of *G. rhinocerontis* is specialized with a long and narrow extension at the ventral side (Figure 1c). Interestingly, the enlarged or elongated pedicels, which either partially (e.g., *Gasterophilus* species, *H*. *bovis* and *H*. *lineatum*) or totally (e.g., *P*. *magnifica*) enclose the postpedicel, have been reported in many bot fly species in different families [38]. The significantly enlarged pedicel are reported in obligate parasitic flies, such as the mouse warble fly genus *Portschinskia* [34] and the louse fly [45,46], and thus are common characteristics of the subfamilies Gasterophilinae, Oestrinae, and Hypodermatinae (Table 2). These structures were speculated to protect the numerous fragile sensilla on the postpedicel surface from mechanical damage [46,47], but they may also obstruct the olfactory functions of antennae by reducing the chances for the sensilla to interact with odour molecules in the air. The exact function of these paradoxical structures remains to be determined, perhaps by using techniques that can manifest the details of the interaction between airflow and the insects [48].

Our comparative study shows that the olfactory apparatuses on the postpedicel of *G. rhinocerontis* are unique among bot flies, although the types and the general morphology of the sensilla of *G. rhinocerontis* are similar to that of most other Oestridae species, despite their differences in body size and antennal size (Figure 4). The pedicellar button, which is usually observed in the articular surfaces between pedicel and postpedicel in calyptrates [26,51], was not detected in *G. rhinocerontis*. We estimated the number of sensory pits to range from 500–900 per postpedicel, with each containing ~50 sensilla, as other Oestridae species have significantly fewer sensory pits containing fewer sensilla (Table 2). Thus, *G. rhinocerontis* contains surprisingly more olfactory sensilla than any other species in this family. Neurons are one of the most demanding tissues of animals [52], as such a high number of sensilla require a massive amount of energy and resources to develop and maintain. Compared to the rest of the parasitic bot fly species, the antennal size and sensilla number reflect the high selection pressure of acute olfactory functions of *G. rhinocerontis*.

The number of sensory pits in *G. rhinocerontis* stands out among this family, at magnitudes higher than the majority of the Oestridae species (Table 2). Sensory pits may accommodate many more sensilla on antennae of the same size, which may significantly increase the surface area of antennae and hence the ability to capture odour molecules, a typical strategy for fly species requiring highly sensitive olfactory functions. In *G. rhinocerontis*, numerous sensory pits aggregate to form ‘super sensory pits’ on the postpedicel (Figure 3a), which further increase the density of sensilla and presumably enhance the olfactory functions. The exact odours that sensory pits perceive remain largely unknown, presumably due to the difficulty of applying single sensillum recording (SSR) techniques on these structures. The morphological diversity of these structures suggests that they may function differently between species in different clades and with different feeding or oviposition habits (Table 2). Among the Oestridae species, sensory pits may be associated with host allocation, which is extremely important, as the hosts of this family are usually dispersed and fast-moving mammals (Table 2). It may be no coincidence that the two species (*Gasterophilus pecorum* and *Portschinskia magnifica*), from two different subfamilies that oviposit on plant materials, both lack sensory pits, while the other species with sensory pits lay eggs on their hosts.

The antennal arista is regarded as the last article of the postpedicel [49]. It is mainly fringed with sparse microtrichia and is without olfactory sensilla. In *G. rhinocerontis*, several coeloconic sensilla were detected on the arista. Similar arista sensilla were documented in horse stomach bot flies as well (i.e., *Gasterophilus* species) [26], which appear to be a synapomorphy to support the sister-group relationship of *Gyrostigma* and *Gasterophilus*. A long bristle with a pointed apex and long conical base was observed on the arista of male and female specimens (74.67 μm long in female; 76.38 μm long in male).

## 5. Conclusions

Using light and scanning electron microscopy, we thoroughly examined the ultrastructure on the antennae of *Gyrostigma rhinocerontis* for the first time. Only microtrichia and chaetic sensilla were observed on the scape and pedicel, and the latter is enlarged, half-enveloping the postpedicel. Four types of sensilla (trichoid sensilla, basiconic sensilla, coeloconic sensilla, clavate sensilla) and sensory pits were detected on the postpedicel. A set of coeloconic sensilla were located on the arista, which appear to be a synapomorphy to support the sister-group relationship of *Gyrostigma* and *Gasterophilus*. The antennae of *G. rhinocerontis* are the largest among Oestridae species, with the most sensilla and the most sensory pits. These antennal characteristics could serve to improve the perception of volatile stimuli, allowing *G. rhinocerontis* to locate their endangered hosts. The morphological information collected in our study may offer a wealth of information for further investigations of the sensory physiological function of each morphological type of sensilla, as well as insights into the evolutionary history of bot flies, which has valuable potential in phylogenetic studies. The lack of museum collection is an important constraint on the morphological study of Oestridae, mainly because bot flies are often poorly represented in museum collections, except for some species of *Cuterebra* and a few species that parasitise livestock. Due to the limited number of specimens we inspected (using only 1 female and 1 male for SEM work), the morphometric data of antennal sensilla in *G. rhinocerontis* must be interpreted carefully when compared with other bot flies as part of further research.

## Figures and Tables

**Table 1:** Morphometric data of antennal sensilla in *Gyrostigma rhinocerontis* (mean ± SD, *n* = 15, *n* = 1 for Ch II).

Sensilla Type	Sex	Length (μm)	Basal Diameter (μm)	Tip Diameter (μm)
Ch I	Female	133.10 ± 4.28	−	−
	Male	−	−	−
Ch II	Female	74.67	−	−
	Male	76.38	−	−
Tr	Female	18.15 ± 1.82	2.14 ± 0.32	−
	Male	18.05 ± 0.59	2.26 ± 0.15	
Ba I	Female	11.33 ± 1.99	2.40 ± 0.42	−
	Male	10.05 ± 0.47	2.46 ± 0.30	
Ba II	Female	7.85 ± 0.61	2.94 ± 0.63	−
	Male	−	−	−
Co I	Female	2.88 ± 0.40	1.97 ± 0.25	−
	Male	2.59 ± 0.13	1.92 ± 0.13	−
Co II	Female	6.87 ± 0.58	2.35 ± 0.12	−
	Male	6.54 ± 0.32	2.23 ± 0.15	−
Cl	Female	8.29 ± 0.94	2.01 ± 0.14	2.20 ± 0.15
	Male	7.92 ± 0.52	2.10 ± 0.30	2.21 ± 0.14

Ba I = basiconic sensilla I; Ba II = basiconic sensilla II; Ch I = chaetic sensilla I; Ch II = chaetic sensillum II; Cl = clavate sensilla; Co I = coeloconic sensilla I; Co II = coeloconic sensilla II; Tr = trichoid sensilla I; F = female; − = undetermined.

**Table 2:** Comparison of biology, antennal morphology, and antennal ultrastructure in Oestridae. (The data in this table refer to the literature cited after the species name).

Subfamily	Species	Pedicel Shape	Number of Sensory Pits	Sensilla Number in Each Sensory Pit	Host	Oviposition Location
Gasterophilinae	*Gasterophilus haemorrhoidalis* (Linnaeus) [9,26,38]	partly enveloping postpedicel	60	2–5 (Auriculate sensilla)	Equine	Singly on hair of lips (on host)
	*Gasterophilus intestinalis* (De Geer) [9,26,38]	partly enveloping postpedicel	83	2–5 (Auriculate sensilla)	Equine	Hairs on forelegs and chest (on host)
	*Gasterophilus nasalis* (Linnaeus) [9,26,38]	partly enveloping postpedicel	47	1 (Clavate sensilla)	Equine	Hairs under chin (on host)
	*Gasterophilus pecorum* (Fabricius) [9,26,38]	partly enveloping postpedicel	0	0	Equine	Grass or vegetation
	*Gasterophilus nigricornis* (Loew) [9,25,38]	partly enveloping postpedicel	68	5–7 (Auriculate sensilla)	Equine	Hairs of cheek or neck (on host)
	*Gyrostigma rhinocerontis* (Hope) [9,26,38]	greatly enlarged and has a reduced ventral part	500–700	~50	Rhinoceros	Skin surface (on host)
	*Cobboldia loxodontis* Brauer [9,38]	partly enveloping the postpedicel	unknown	unknown	Elephants	Base of tusk (on host)
Oestrinae	*Oestrus ovis* Linnaeus [9,38,49]	partly enveloping the postpedicel	50	3 (Basiconica sensilla)	Ovine	All Oestrinae are larviparous; forcefully inject their progeny on to host
	*Rhinoestrus purpureus* (Brauer) [9,28,38]	partly enveloping the postpedicel	37	2–8 (Clavate sensilla; basiconic sensilla)	Equine	(Same as above)
Hypodermatinae	*Hypoderma bovis* (Linnaeus) [9,24,38]	surrounding and enclosing base of postpedicel	>300	<6 (Basiconica sensilla)	Bovine	Hairs of host
	*Hypoderma lineatum* (Villers) [9,27,38]	enlarged and encircles nearly entire postpedicel	30	2–6 (Basiconic sensilla; Trichoid sensilla)	Bovine	Hairs of host
	*Oestromyia leporina* (Pallas) [9,38], unpublished data	partly enveloping postpedicel	25	2–5	Pikas	Hairs of host
	*Portschinskia magnifica* Pleske [9,34,38]	hollowed pedicel entirely enclosing postpedicel	0	-	Mice	unknown
Cuterebrinae	*Dermatobia hominis* (Linnaeus Jr.) [8,38,50]	Normal shape as in other calyptrate flies	~3–5	1 (Coeloconic sensilla)	Non-specific; larger mammals and birds	Egg attached to different species of blood-feeding insects captured by female bot fly.

## Data Availability

The data generated in this study are provided here and they are also available upon request from the corresponding author.

## Supplementary Materials

The following supporting information can be downloaded at: www.mdpi.com/article/10.3390/insects13100889/s1, File S1: Habitus of the entire fly of *Gyrostigma rhinocerontis* (female).
