# Supplementary material for: Insights into the Antennal Characteristics and Olfactory Strategy of the Endangered Rhino Stomach Bot Fly Gyrostigma rhinocerontis (Diptera: Oestridae)
Source: Insects. 2022 Sep 29;13(10):889. doi: 10.3390/insects13100889 (PMC9604252; doi:10.3390/insects13100889)
Supplement: Supplementary file 1 [file insects-13-00889-s001.zip › insects-1911715-supplementary.pdf]

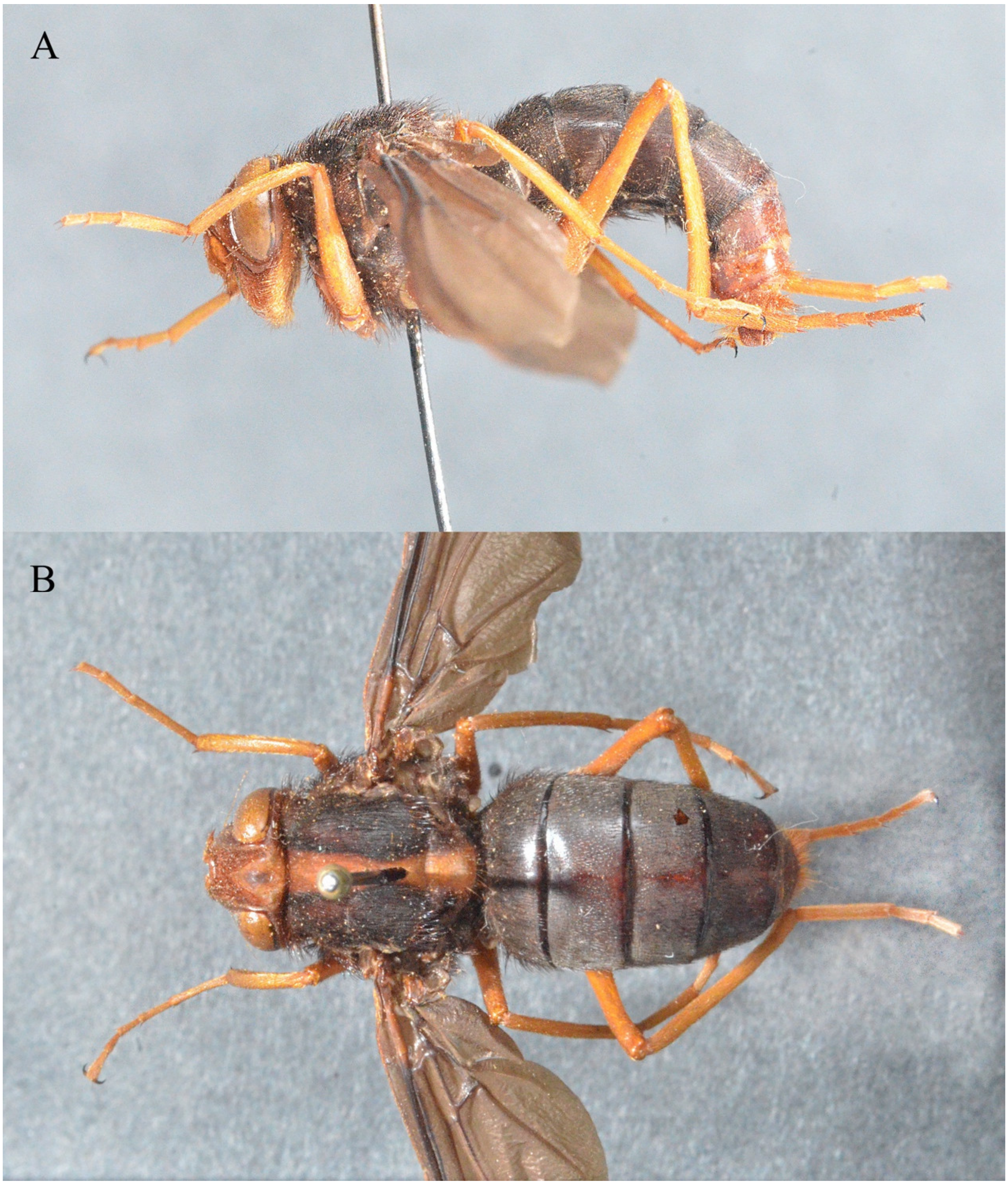

Supplementary File S1. Habitus of the entire fly of *Gyrostigma rhinocerontis* (female). (**A**) Habitus, lateral view; (**B**) Habitus, dorsal view.
